# Supplementary material for: MetaQTL: a package of new computational methods for the meta-analysis of QTL mapping experiments
Source: BMC Bioinformatics. 2007 Feb 8;8:49. doi: 10.1186/1471-2105-8-49 (PMC1808479; doi:10.1186/1471-2105-8-49)
Supplement: Additional File 7 — MetaQTL Package : jar file and tutorial. This Zip archive contains both the MetaQTL JAR file and the files of the tutorial. [file 1471-2105-8-49-S7.zip › org.inra.metaqtl/doc/org/metaqtl/package-summary.html]

org.metaqtl


|  |  |  |
| --- | --- | --- |
| **Overview** \| **Package** \| Class \| **Use** \| **Tree** \| **Deprecated** \| **Index** \| **Help** | |  |
| PREV PACKAGE   **NEXT PACKAGE** | **FRAMES**    **NO FRAMES**     **All Classes** |  |


---

## Package org.metaqtl

| **Interface Summary** | |
| --- | --- |
| **IBioGenomeProperties** | This interface defines both the keys and the default values of properties which can be handled by a org.thalia.bio.entity.IBioGenome. |
| **IBioLGroupProperties** | This interface defines the key of properties which can be handled by a org.thalia.bio.entity.IBioLGroup. |
| **IBioLocusProperties** | This interface defines the key of the properties which can be handled by a org.thalia.bio.entity.IBioLocus. |
| **IMetaAlgorithm** | This interface defines some methods which are common to data analysis algorithms. |
| **IMetaQtlConstants** | This interface defines some global constants which are used by several classes of the package. |

| **Class Summary** | |
| --- | --- |
| **ChromCluster** | A cluster of chromosomes which share the same name inside a `MapCluster` framwork. |
| **Chromosome** | On a Chromosome there are two types of locus : Marker QTL (Quantitative Trait Loci) inside a mapping context which is defined by both the mapping framework (function and unit) and the population cross design used to build this chromosome. |
| **CMarkerSequence** | A Common marker sequence between two ordered set of markers is a sub-set of at least two markers which order is the same between the two sets (may be reverse). |
| **EMCriteria** |  |
| **EMResult** |  |
| **MapCluster** |  |
| **MetaChrom** | A MetaChrom is the result of the merge of the members of a `ChromCluster`. |
| **MetaDico** | This class represents a dictionnary of synonyms. |
| **MetaMap** | A `MetaMap` is a bundle of `MetaChrom` objects. |
| **MetaQtlAnalysis** | A QTL meta analysis consists in a clustering of QTL positions over chromosomes for different group of traits. |
| **MetaQtlData** | QTL meta analysis first requires to gather the QTL according to the group trait they belong and then to format the QTL data into data points to perfom clustering. |
| **MetaQtlModel** |  |
| **MetaQtlResult** | A `MetaQtlResult` is used to store the results of a QTL meta analysis which has been performed for a given trait group on a given chromosome. |
| **MultiFitResult** | This class represents a result of a multilinear regression analysis. |
| **Qtl** | A QTL (Quantitative Trait Loci) |
| **QtlPartition** |  |
| **Tree** | This class defines a tree. |
| **TreeNode** | This class represents a node in a binary Tree obtained when applying usual hirarchical clustering methods. |

---


|  |  |  |
| --- | --- | --- |
| **Overview** \| **Package** \| Class \| **Use** \| **Tree** \| **Deprecated** \| **Index** \| **Help** | |  |
| PREV PACKAGE   **NEXT PACKAGE** | **FRAMES**    **NO FRAMES**     **All Classes** |  |


---
